# A case report of a recurrent early and late Bioresorbable vascular scaffold thrombosis: serial angiography and optical coherence tomography findings

**DOI:** 10.1186/s12872-020-01426-z

**Published:** 2020-03-24

**Authors:** Cheol Hyun Lee, Yun-Kyeong Cho, Hyuck-Jun Yoon, Seung-Ho Hur

**Affiliations:** Division of Cardiology, Keimyung University Dongsan Medical Center, 56 Dalseong-Ro, Jung-Gu, Daegu, 700-712 South Korea

**Keywords:** Bioresorbable vascular scaffold, Scaffold thrombosis, Optical coherence tomography, Acute coronary syndromes

## Abstract

**Background:**

In bioresorbable vascular scaffolds (BVSs), there is some concern about a possible increase in the rate of scaffold thromboses (ScTs). Although several characteristics similarly contribute to the development of both early and late ScTs, there are also clearly different pathomechanisms between the two time-dependent types of thromboses, especially with BVSs.

**Case presentation:**

We recently experienced a very rare case of a 69-year-old man who had recurrent early and late ScTs with somewhat differing pathomechanisms as assessed by optical coherence tomography (OCT). For the late ScT, OCT identified a scaffold dismantling in the same place that a peri-strut low intensity area (PLIA) was observed in the previous OCT finding.

**Conclusion:**

We report the management of an ScT in a case with findings such as a heterogeneous a BVS degradation, peri-strut low intensity area (PLIA), intraluminal scaffold dismantling, and under-sizing and/or stent malapposition observed in OCT.

## Background

A stent thrombosis (ST) is an uncommon but devastating adverse event that can occur after percutaneous coronary intervention (PCI) [1]. Various factors can influence the likelihood of an ST occurrence, including the patient-related characteristics, procedural and lesion characteristics, presence of acute coronary syndrome, and premature discontinuation of antiplatelet therapy [2, 3]. With the advent of newer-generation drug-eluting stents (DES) and the continuation of a dual antiplatelet therapy (DAPT), this phenomenon has come to be seen less frequently. However, with bioresorbable vascular scaffolds (BVSs), there is some concern about an increased rate of scaffold thromboses (ScTs) [4]. Optical coherence tomography (OCT) imaging is instrumental in identifying the mechanisms of STs as well as ScTs [5]. Several case reports have revealed the mechanism of ScTs using OCT. Here, we describe and discuss a rare recurrent ScT case with an intraluminal BVS dismantling identified by OCT, which may have led to a very late thrombosis in a patient with a history of a subacute ScT, who presented with acute coronary syndrome 23 months after the BVS placement.

## Case presentation

**Fig. 1:**
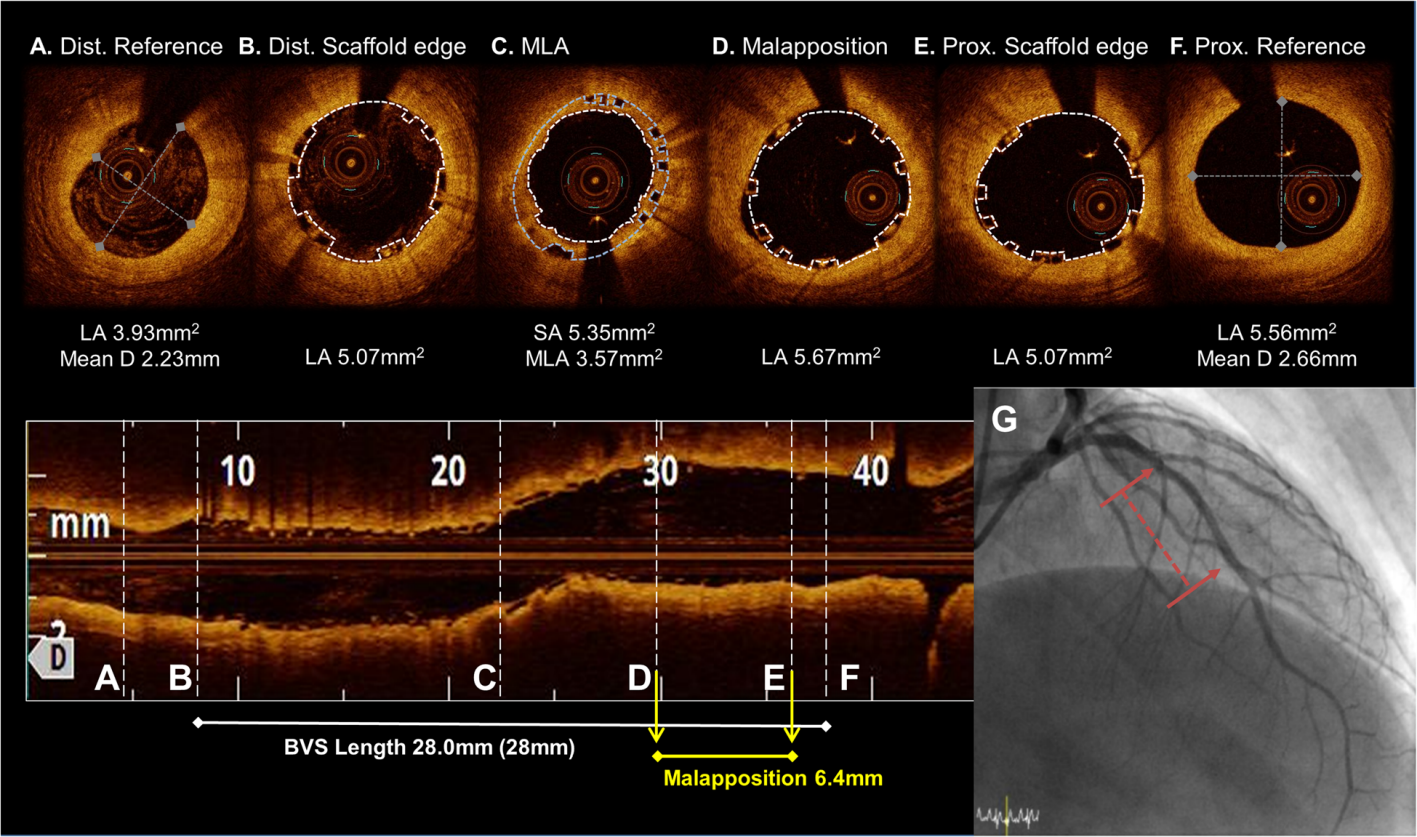
The OCT images during the BVS implantation from the distal to proximal 2.5 × 28 mm BVS was deployed at a nominal pressure and post-dilated with a 2.75 mm noncompliance balloon at 16 atm. (**a to f**). An under-sizing and minimal malapposition over the cutoff value (< 200 μm) were seen at the proximal part of the BVS. (**c to e**) A tissue prolapse was seen at the MLA site. (**c**) The final coronary angiogram at baseline following the BVS implantation (red dotted line). (**g**) Abbreviations: Dist., distal; Prox., proximal; MLA, minimal lumen area; LA, lumen area; D, diameter; SA, scaffold area; BVS, bioresorbable vascular scaffolds

**Fig. 2:**
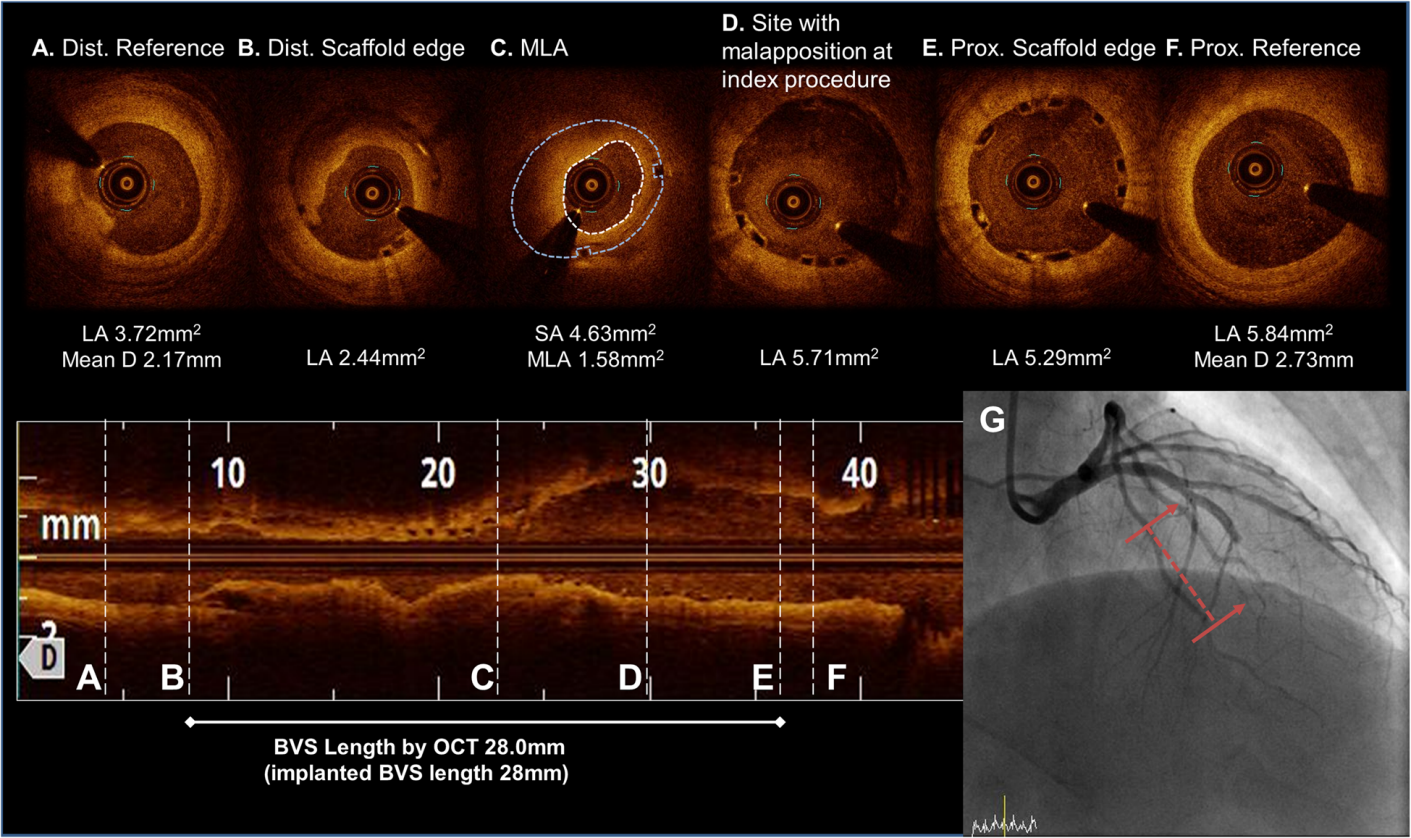
OCT image during the subacute scaffold thrombosis event after a 2.5 mm preballoon dilatation up to 4 atm. (**a to f**). Intraluminal red thrombus with stacked struts in the middle to distal part of the BVS. (**b to c**) No previous under-sizing and malapposition could be seen in the proximal part of the BVS. (**c to e**) Coronary angiogram of a subacute scaffold thrombosis event. A TIMI 0 flow was observed in the distal part of the previous BVS (red dotted line). (**g**) Abbreviations: Dist., distal; Prox., proximal; Pre-, previous; MLA, minimal lumen area; LA, lumen area; D, diameter; SA, scaffold area; BVS, bioresorbable vascular scaffolds

**Fig. 3:**
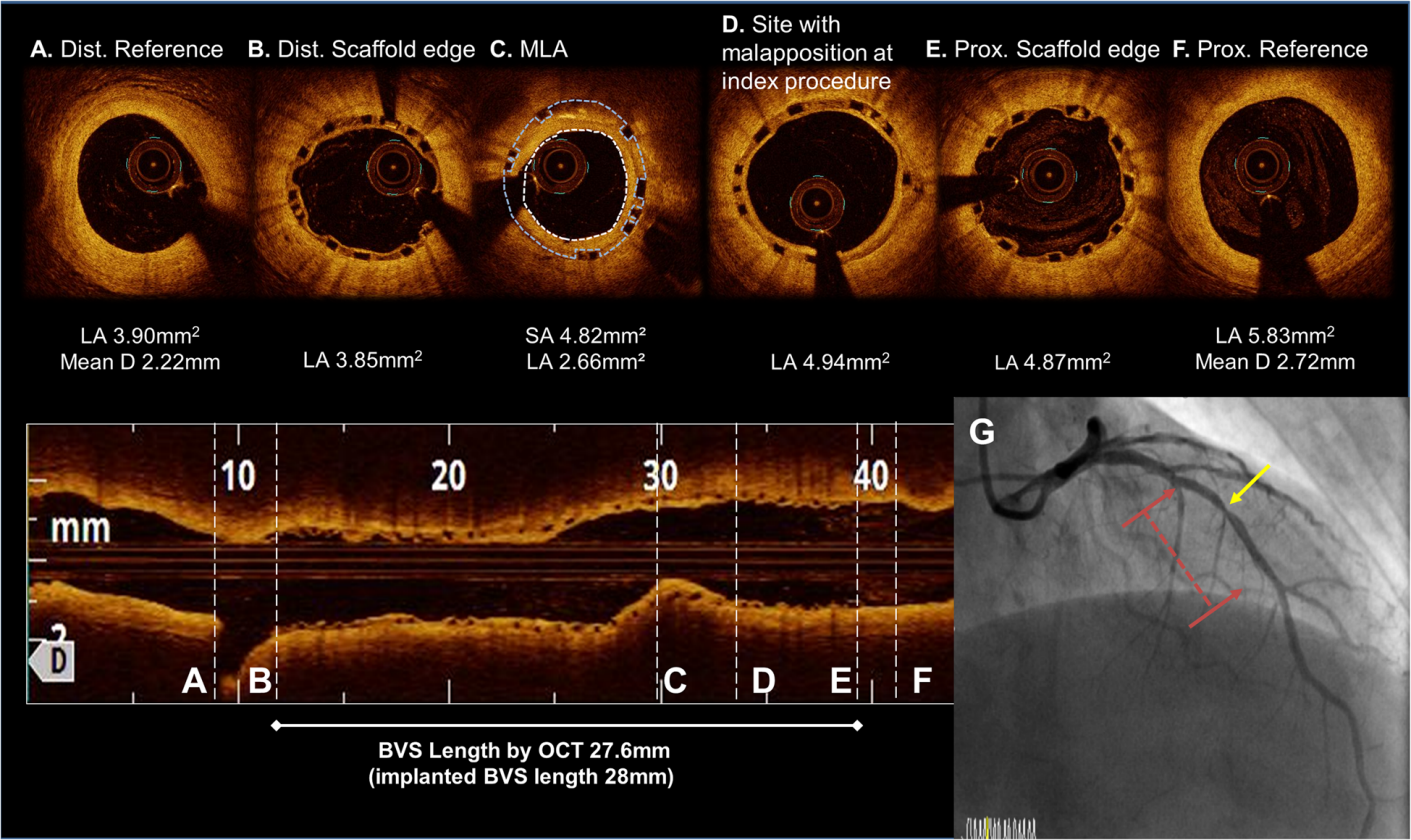
OCT image at the one-year follow up after the BVS implantation. (**a to f**). The MLA site migrated to the proximal side of the BVS. (**c**) A peristrut low intensity area (PLIA) was observed in the proximal part of the BVS. (**d**) A coronary angiogram at the one-year follow up after the BVS implantation. A discrete moderate stenosis was observed at the MLA site (yellow arrow) of the previous BVS. (red arrow line). (**g**) Abbreviations: Dist., distal; Prox., proximal; Pre-, previous; MLA, minimal lumen area; LA, lumen area; D, diameter; SA, scaffold area; BVS, bioresorbable vascular scaffolds

A 69-year-old man, a current smoker with hypertension and diabetes mellitus, presented to the emergency department with a non-ST-segment elevation myocardial infarction (NSTEMI). Coronary angiography revealed a total occlusion of the mid left anterior descending artery (LAD). He underwent a placement of a 2.5 × 28 mm Absorb GT1 BVS (Abbott Vascular, Santa Clara, CA, USA) under OCT-guidance, pre-dilated with a 2.0 × 20 mm TREK (Abbott Vascular, Santa Clara, CA) balloon and post-dilated with a 2.75 × 15 mm non-compliant (NC) TREK balloon up to 12 atm in his mid-LAD (Fig. 1). The OCT images after the initial scaffold implantation showed an acute malapposition of 6.4 mm in length at the proximal part of the BVS, likely due to the use of an undersized BVS. After high pressure ballooning, it no longer exhibited a significant value (< 200 μm) on OCT. He was prescribed aspirin and clopidogrel upon discharge, but arbitrarily stopped both for a colonoscopy 16 days later. He was re-admitted to the hospital with chest pain 20 days after the BVS implantation. An NSTEMI was confirmed by a subacute ScT in the middle of the scaffold on the coronary angiography examination. We performed a pre-dilation at the site of the scaffold using a 2.5 × 20 mm semi-compliant balloon and angioplasty with a 3.0 × 12 mm NC balloon up to 18 atm in order to obtain a TIMI 3 flow (Fig. 2). He was prescribed aspirin and ticagrelor upon re-discharge. He showed no abnormalities other than mild stenosis in the middle of the scaffold at the one-year follow-up coronary angiography. The one-year follow up OCT (Dragonfly, St. Jude Medical, St. Paul, MN, USA) revealed a peri-strut low intensity area (PLIA) in the proximal part of the BVS. However, since the minimal lumen area (MLA) was 2.66 mm^2^ and the neointimal hyperplasia (NIH) was 55% without symptoms, we decided to continue medication (Fig. 3). Since an acute myocardial infarction occurred at 1 year, aspirin and clopidogrel were prescribed again.

**Fig. 4:**
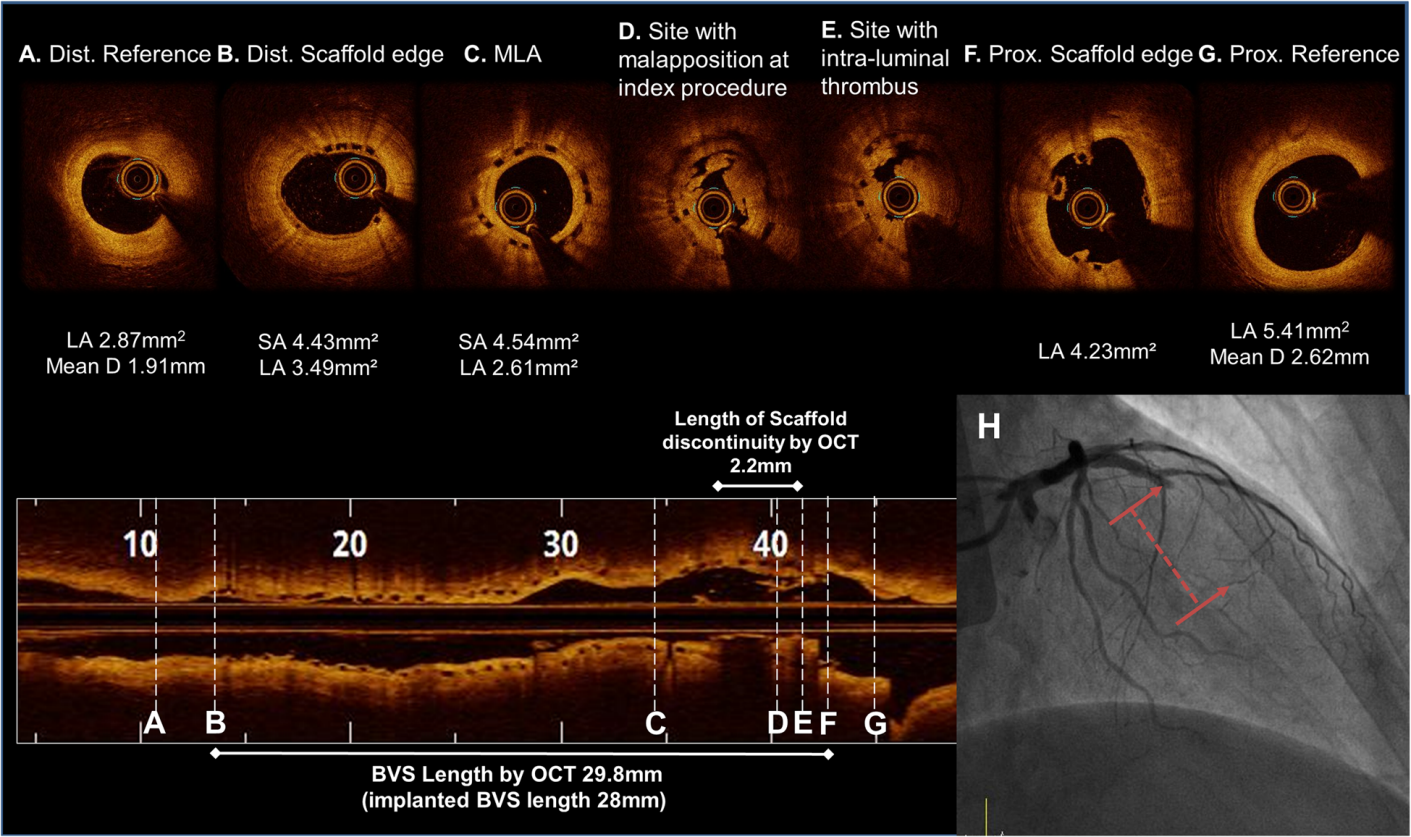
OCT image during the very late scaffold thrombosis (VLScT) event after a 2.0 mm preballoon dilatation up to 4 atm (**a to g**). The thrombus was observed at the same site where the peri-strut low intensity area (PLIA) had been previously observed, and the strut had been dismantled and was floating in the intraluminal area. (**d to e**) In the coronary angiogram during the VLScT event, a TIMI 0 flow was observed in the proximal part of the previous BVS (red dotted line). (**h**) Abbreviations: Dist., distal; Prox., proximal; Pre-, previous; MLA, minimal lumen area; LA, lumen area; D, diameter; SA, scaffold area; BVS, bioresorbable vascular scaffolds

**Fig. 5:**
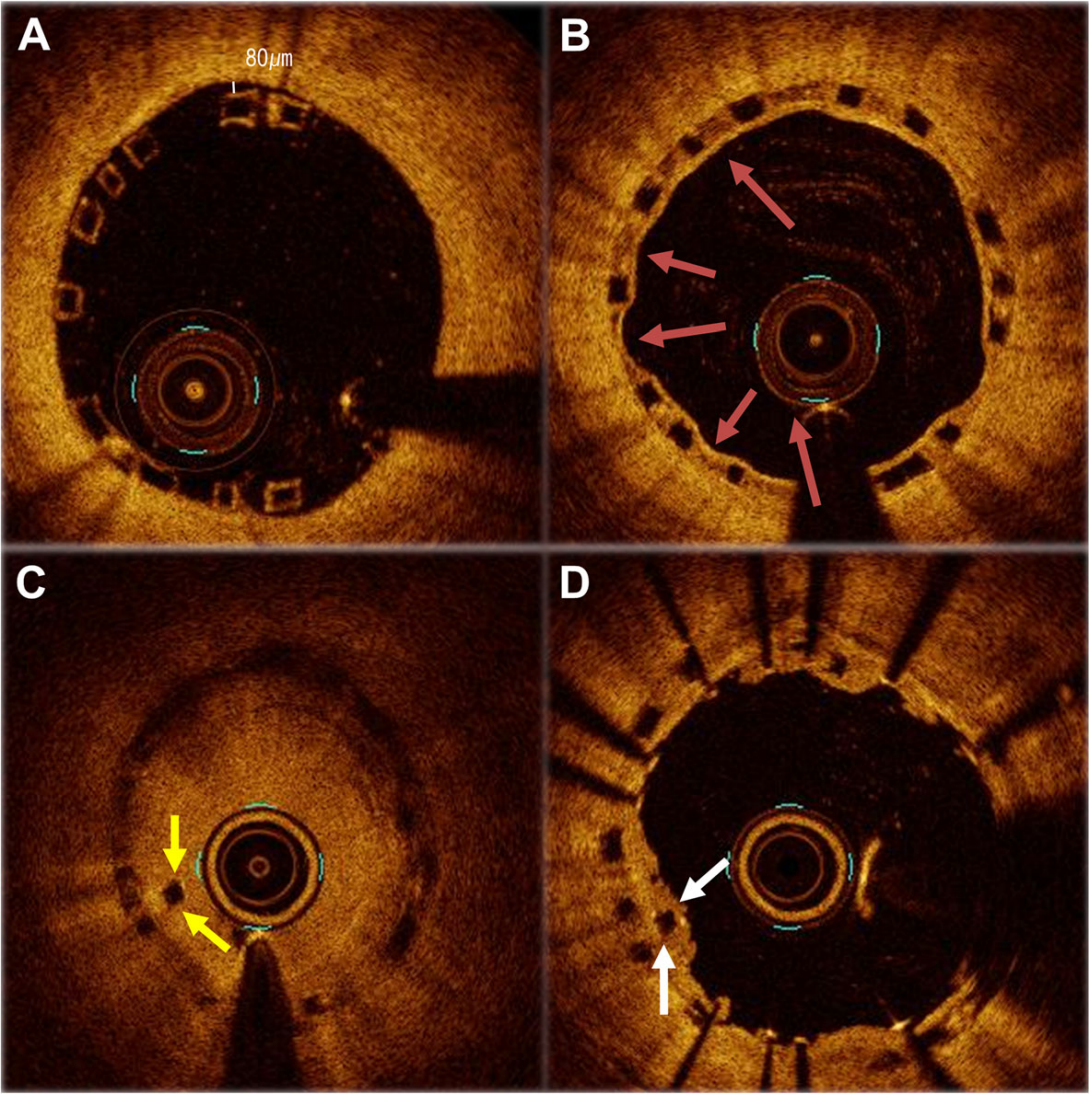
An expanded serial OCT image of the same point during the initial procedure, the one-year follow up, and very late scaffold thrombosis (VLScT) event. During the initial procedure, a minimal malapposition after high pressure NC ballooning is observed. (**a**) A PLIA was observed in the proximal part of the BVS at the one-year follow up (red arrow). (**b**) The thrombus was observed at the site where the PLIA was observed, and the strut had been dismantled and was floating in the intraluminal area during the VLScT event (yellow arrow). This image obtained before balloon predilatation. (**c**) At the time of the VLScT, the Xience alpine 2.75 × 33 mm stent covered the inside of the disrupted scaffold strut with compression toward the vessel wall (white arrow), and a good-expansion was observed in the final OCT image. (**d**)

Two years after the BVS implantation, the patient was referred to the hospital for chest pain at rest. The patient underwent coronary angiography because the patient developed an ST-segment elevation myocardial infarction (STEMI). Coronary angiography revealed a very late ScT in the proximal part of the prior BVS in the LAD artery (Fig. 4). OCT identified a thrombus in the proximal segment of the scaffold, at the same place where the PLIA was observed in the previous OCT finding. Two layers of the BVS struts, consisting of a strut supporting the vessel wall and a strut protruding into the lumen, were also detected (Fig. 5). Since the BVS struts were disrupted with an increased risk of a distal embolization by an aspiration catheter, direct balloon angioplasty was performed immediately without a thrombus aspiration in order to obtain coronary flow. A 2.75 × 33 mm Xience Alpine balloon (Abbott Vascular, Santa Clara, CA) was then deployed at 10 atm in the LAD covering the whole segment of the prior scaffold. The stent was then post-dilated with a 3.0 × 12 mm non-compliant balloon at 16 atm. The entire newly-placed DES showed a good apposition, as the intra-luminal dismantled BVS struts were compressed toward the vessel wall, according to the OCT examination.

## Discussion and conclusions

The occurrence of a stent thrombosis after a metallic DES implantation has been attributed to inflammatory and allergic reactions to specific clinical and stent components (clinical presentation, drug, polymer, or stent). In contrast, a scaffold thrombosis has been reported to be caused by various factors beyond the factors of a metallic DES. Our case had important implications for the OCT assessment when evaluating the underlying pathomechanisms of early and late ScTs.

The key factor of the early and subacute ScT in the present case may have been the discontinuation of the dual antiplatelet therapy (DAPT). Although some mechanical factors such as a malapposition may have contributed to the development of the ScT, when the subacute ScT event occurred, a significant acute malapposition (≥ 200 μm) was no longer observed on OCT after an adjunctive NC balloon inflation. Therefore, we decided to use a more potent P2Y12 inhibitor after ensuring coronary flow by balloon angioplasty as opposed to an additional DES implantation. Maintaining a DAPT beyond 1 year is more important than a metallic DES for the prevention of a late and very late ScT with BVSs, because the degradation of a BVS strut can heterogenically occur even over 3 to 4 years [6]. In addition, our case exhibited a procoagulable status under the NSTEMI condition. The patient had to maintain the DAPT for over a year according to the guidelines for preventing future events [7]. Several reports have identified under-sizing as a factor for an early ScT with both DESs and BVSs [8–11]. In the case shown in Fig. 1, the implantation of an undersized BVS may have been another mechanism of the subacute ScT, because a 2.5 mm scaffold was implanted in a vessel with an external elastic lamina (EEL) diameter of approximately 3.0 mm. However, unsurprisingly, patients with under-sizing are likely to be treated for an acute myocardial infarction (AMI) during the index procedure, because there are elevated potent vasoconstrictors in the blood level as well as the presence of microcirculatory dysfunction in the setting of an AMI [12, 13]. In our case, despite concern for under-sizing, the minimal scaffold area in the index procedure was not small (> 5 mm^2^) after post-NC ballooning up to 12 atm, leading to no need for further intervention.

A delayed inflammatory reaction has been identified as a possible mechanism associated with inflammation of late/very late ScTs following the implantation of a BVS. A PLIA might be a marker of vascular edema and vascular vulnerability by previous OCT study because of the degradation products of poly-lactic acid crystals, the substrate for a proteoglycan rich matrix [14]. Considering our case based on previous research, 23 months after the BVS implantation, the BVS strut showed intraluminal dismantling and strut disruption in the same segment where the PLIA had been seen in the one-year follow-up OCT. Eventually, the remaining minimal malapposition increased the vulnerability through the inflammation mechanism and vascular edema in the late reabsorbtion process. The PLIA finding as an unhealthy cover of the BVS strut consequently might not be able to prevent a strut disruption. It became the possible factor of a very late scaffold thrombosis (VLScT). Furthermore, a thick strut with a minimal malapposition may provide high shear stress arising from an incompletely apposed and bulky stent strut within the proximal transition zone. This may have also contributed to the occurrence of a late/very late ScT. A recent report of OCT findings in four cases of VLScTs suggests that discontinuity of the scaffold may cause a VLScT in advanced stages of a scaffold resorption, although the possibility of scaffold damage by a thrombus aspiration procedure could not be ruled out [15]. However, our OCT images were obtained before (Fig. 5c) and after the inflation of a small sized balloon with a low pressure (Fig. 4). Thus, it was important to confirm that the scaffold was dismantled intraluminally during the late period, not due to scaffold damage by the thrombectomy.

We found that a disruption of the strut of the VLScT was a possible nidus through the OCT. We treated it with a long DES adequate to cover all previously-implanted scaffolds. We changed the P2Y12 inhibitor back to ticagrelor, a more potent P2Y12 inhibitor, because we observed a higher level of platelet reactivity in the platelet function test or verify now test when we experienced the recurrent ScT. In addition, as mentioned above, the long-term maintenance of the DAPT beyond 1 year may be helpful, because the degradation of the BVS strut has heterogenicity. In summary, if the occurrence of an ScT is caused by a DAPT interruption, under-expansion, or under-sizing during the acute and subacute periods, a thrombectomy and balloon angioplasty can be considered. If a scaffold fracture and geographical miss are observed in the long segment during the late and very late periods, DES treatment can be performed [16, 17].

Overall, OCT plays a major role in cases of a BVS late thrombosis in identifying associated conditions such as a PLIA, accurate evaluation of a luminal stenosis arising from intimal hyperplasia, intraluminal scaffold dismantling, under-sizing, and/or stent malapposition. On the other hand, these findings, which can only be seen on OCT, may increase the ScT rate, which may eventually be the reason why it cannot be used in real practice. The limitation of this case report is that OCT findings cannot explain all causes of the 2nd ScT event. For this patient, not only the occurrence of scaffold discontinuity associated with a PLIA on OCT, but also the flow disturbance of the MLA site and the patient’s clopidogrel resistance may be involved in the 2nd ScT event. However, our case report showed a hypothesis generating role in which patients with a BVS implantation should actively analyze the OCT findings in order to prevent or identify the cause of the ScT. In addition, further studies are required to define the natural course of a PLIA following a BVS implantation. A next generation bioresorbable scaffold that can minimize late inflammation may need to be developed.

In conclusion, we report a unique case of a subacute and very late scaffold recurrent thrombosis likely due to the withdrawal of antiplatelet agents and late scaffold dismantling, which led to acute coronary syndrome. A serial OCT examination can illustrate the pathophysiologic process during the development of a recurrent ScT. This report emphasizes the importance of OCT-guided treatment planning in the event of problems following a BVS implantation. It may be used as a reference for the future development of a newer BVS device.

## Data Availability

The data analyzed in the case report (medical history of patient) are not publicly available due to the privacy policy of the hospital but are available from the corresponding author on reasonable request.

## Abbreviations

BVS: Bioresorbable vascular scaffolds
DAPT: Dual antiplatelet therapy
DES: Drug-eluting stents
EEL: External elastic lamina
LAD: Left anterior descending artery
MLA: Minimal lumen area
NC: Non-compliant
NIH: Neointimal hyperplasia
NSTEMI: Non ST-segment elevation myocardial infarction
OCT: Optical coherence tomography
PLIA: Peri-strut low intensity area
ScT: Scaffold thrombosis
ST: Stent thrombosis
STEMI: ST-segment elevation myocardial infarction
VLScT: Very late scaffold thrombosis
